# Effectiveness and safety of Shengxuening for treatment of renal anemia: a comprehensive systematic review and meta-analysis

**DOI:** 10.3389/fphar.2025.1510227

**Published:** 2025-06-24

**Authors:** Li Zheng, Xiaotong Gu, Ming Liu, Changhai Fu, Yan Wang

**Affiliations:** ^1^ Department of Pharmacy, China Aerospace Science and Industry Corporation 731 Hospital, Beijing, China; ^2^ Evidence-Based Medicine Center, School of Basic Medical Sciences, Lanzhou University, Lanzhou, China; ^3^ Department of Nephrology, China Aerospace Science and Industry Corporation, Beijing, China; ^4^ Department of Nephrology, China Aerospace Science and Industry Corporation 731 Hospital, Beijing, China

**Keywords:** renal anemia, Shengxuening, systematic review, meta-analysis, GRADE

## Abstract

**Aim:**

We aimed to evaluate the efficacy and safety of Shengxuening (SXN) in treating renal anemia by systematic review and meta-analysis.

**Methods:**

PubMed, Embase, the Cochrane Library, the ClinicalTrials.gov, SinoMed, the China Knowledge Network, the Wanfang Data Knowledge Service Platform and Technology Journal Database were searched from inception to September 2024 randomized controlled trials (RCTs) that compared SXN and other drugs or placebo in treating renal anemia. We used the Cochrane Bias Risk Tool to evaluate the risk of bias of all included RCTs, and used the Grading of Recommendations, Assessment, Development and Evaluation (GRADE) to evaluate the certainty of the evidence.

**Results:**

31 RCTs involving 2,372 patients were included. The efficiency of SXN was superior than the control group (included placebo group, other drugs group) in improving hemoglobin (compared with placebo: MD: 7.15 g/L, 95% CI: 5.68–8.62, P < 0.001, GRADE: high; compared with other drugs: MD: 6.49 g/L, 95% CI: 3.50–9.47, P < 0.001, GRADE: very low), serum ferritin (MD: 57.53 ng/mL, 95% CI: 29.70–85.36, P < 0.001, GRADE: moderate; MD: 28.96 ng/mL, 95% CI: 1.88–56.04, P = 0.04, GRADE: moderate) and transferrin saturation level (MD: 7.00%, 95% CI: 3.40–10.60, P = 0.0001, GRADE: moderate; MD: 3.64%, 95% CI: 1.41–5.88, P = 0.001, GRADE: moderate). Besides, the efficiency of SXN combined with other drugs was superior than other drugs group in improving hemoglobin (MD: 11.95 g/L, 95% CI: 6.19–17.71, P < 0.001, GRADE: moderate), serum ferritin (MD: 53.43 ng/mL, 95% CI: 20.65–86.21, P = 0.001, GRADE: moderate) and transferrin saturation level (MD: 5.91%, 95% CI: 3.72–8.10, P < 0.001, GRADE: moderate). Additionally, the incidence of adverse drug reactions (ADRs) in the SXN group was lower than that in other drugs treatment group (OR: 0.20, 95%CI: 0.12–0.33, P < 0.00001).

**Conclusion:**

The efficacy of SXN in treating renal anemia is convincing. Compared with other drugs, SXN is comparable or even better in treating renal anemia. Additionally, the safety of SXN is also relatively high.

## 1 Introduction

Renal anemia is a prevalent and serious complication of chronic kidney disease (CKD) (Haalen et al., 2020). It is strongly correlates with a significant adverse outcome on the burden and progression of the CKD, and has a strong negative impact on quality of life, hospitalization rates, major adverse cardiovascular events and mortality (Palaka et al., 2020; Taderegew et al., 2023; Habas et al., 2023). The causes of renal anemia include erythropoietin (EPO) deficiency, iron deficiency, disorder of iron metabolism in the body, and resistance to the EPO signaling pathway (Haase, 2017). Absolute or relative deficiency of EPO is the main factor leading to anemia.

Shengxuening (SXN) extracted from nontoxic silkworm excrement is an effective heme-like iron supplement which has hematopoiesis effects on patients with renal anemia and is widely used for the treatment of renal anemia (Ding et al., 2019; Zeng et al., 2020). Its constituents, sodium iron chlorophyllin, can improve iron metabolism, stimulate erythropoiesis in the bone and protect human cells from oxidative stress (Ding et al., 2019). Some clinical studies have indicated that SXN was more effective than oral iron supplementation in the treating renal anemia (Zeng et al., 2020; Zhang L. et al., 2016). The reported of Cheng X et al. (Cheng et al., 2016) showed that SXN combined with EPO was safe and effective in patients with renal anemia.

In the past few years, there has been a growing body of clinical evidence supporting the beneficial effects of SXN in the treatment of renal anemia. However, there is currently a lack of a meta-analysis that combines and analyzes all the available randomized controlled trials (RCTs). So we conducted a systematic review and meta-analysis to explore the effectiveness and safety of SXN in treating renal anemia, and provide evidence for the treatment of renal anemia patients.

## 2 Methods

This meta-analysis was preferred according to the Preferred Reporting Items for Systematic Review and Meta-analysis (PRISMA) and Cochrane Handbook (Moher et al., 2009). We registered the review prospectively in Open Science Frameworks, https://osf.io/qryds/.

### 2.1 Search strategy

We searched PubMed, Embase, the Cochrane Library, the ClinicalTrails.gov, CNKI, Wanfang, VIP and SinoMed from inception to 24 April 2024, without language restrictions. The searched keywords and Medical Subject Headings were “shengxuening,” “sheng xue ning tablets,” “renal anemia” and “chronic kidney disease.” The detailed search strategy of all databases can be seen in Supplementary Text S1.

### 2.2 Eligibility criteria

We included studies in this meta-analysis if they met the following eligibility criteria: 1) the patients with renal anemia who have clear diagnostic criteria; 2) the intervention group used SXN; 3) reported change of one or more of the following outcomes of interest: hemoglobin (Hb), serum ferritin (SF), transferrin saturation (TSAT), serum iron (SI), and adverse drug reactions (ADRs); 4) the study type is a RCT.

We exclude studies without clear diagnostic criteria and inability to obtain the full text. Meanwhile, duplicate articles were excluded.

### 2.3 Study selection

All study search records were imported into the EndNote software. Two authors (XTG and ML) independently screened studies based on the title and abstract, and deleted the articles that did not meet the inclusion criteria. The remaining articles need to be read in full to determine if they are suitable for inclusion in the analysis. When two authors have different opinions, they can resolve it through discussion or have a third author (YW) make the final decision.

### 2.4 Data extraction

The information we screened includes: study title, first author’s name, publication time, study type, patient characteristics, intervention measures, treatment duration, and the outcomes of interest.

Two authors (LZ and XTG) independently extracted data using a standardized form, and in case of disagreements, discussions were held or a decision was made by a third author (CHF). When encountering multiple reports on the same study (such as original full text, conference, abstract, and post-analysis), we will collect all relevant data and analyze it as a study.

### 2.5 Risk of bias assessment

Two authors (LZ and XTG) independently performed the risk of bias of the included RCTs by using the Cochrane Risk of Bias Tool 2.0 (ROB 2) (Sterne et al., 2019) based on based on six domains: bias arising from the randomization process, bias due to deviations from the intended intervention, bias due to missing outcome data, bias in measurement of the outcome, bias in selection of the reported result, and other biases. We sub-classified “some concerns” judgements as “some concerns, probably high” and “some concerns, probably low,” if at least one domain was high, we considered the study at high risk of bias. Any discrepancies were resolved through mutual consultation or determined by a third author (ML).

### 2.6 Statistical analysis

We used software Review Manager (RevMan), version 5.4.1 (The Cochrane Collaboration Network, 2020) to conduct meta-analysis of the included studies. The dichotomous outcomes were evaluated by odds ratio (OR) while the mean difference (MD) was used to represent continuous outcomes, with 95% confidence interval (CI). I^2^ was used to evaluate heterogeneity among included RCTs, and when I^2^ is greater than 50%, it indicates at least moderate heterogeneity between included RCTs. Based on the assumption of significant clinical heterogeneity, a random effects model was used in this meta-analysis. In order to effectively avoid heterogeneity between included RCTs, we divided them into different subgroups for analysis based on the comparison measures of the included studies. We also conducted meta-regression to clarify the source of heterogeneity. Additionally, sensitivity analysis was used to evaluate the stability of the results.

### 2.7 Publication bias

According to the Cochrane Reviewer’s Handbook, Egger’s test require more than 10 studies to be meaningful. Otherwise, their test accuracy decreases, and the assessment of publication bias becomes inaccurate. Therefore, we performed the Egger’s test on the outcomes which was reported at least 10 RCTs by using the statistical software Stata (version 18.0; StataCorp, College Station, TX) to assess potential publication bias, and also used these tests to identify outliers (Egger et al., 1997).

### 2.8 Certainty of evidence

We used the Grading of Recommendations, Assessment, Development and Evaluation (GRADE) approach to assess the certainty of the evidence for the critical outcomes, considering factors such as the risk of bias, imprecision, indirectness, inconsistency and publication bias. The certainty of evidence was graded into four level: (1) very low, any estimate of effect is very uncertain; (2) low, further research is very likely to have a significant impact on our faith in the assessment of development and is expected to change the assessment; (3) moderate, further research is likely to have a significant impact on our faith in the assessment of effect and may change the estimate; (4) high, further research is improbable to change our confidence in the estimate of effect.

## 3 Results

### 3.1 Search results

**FIGURE 1:**
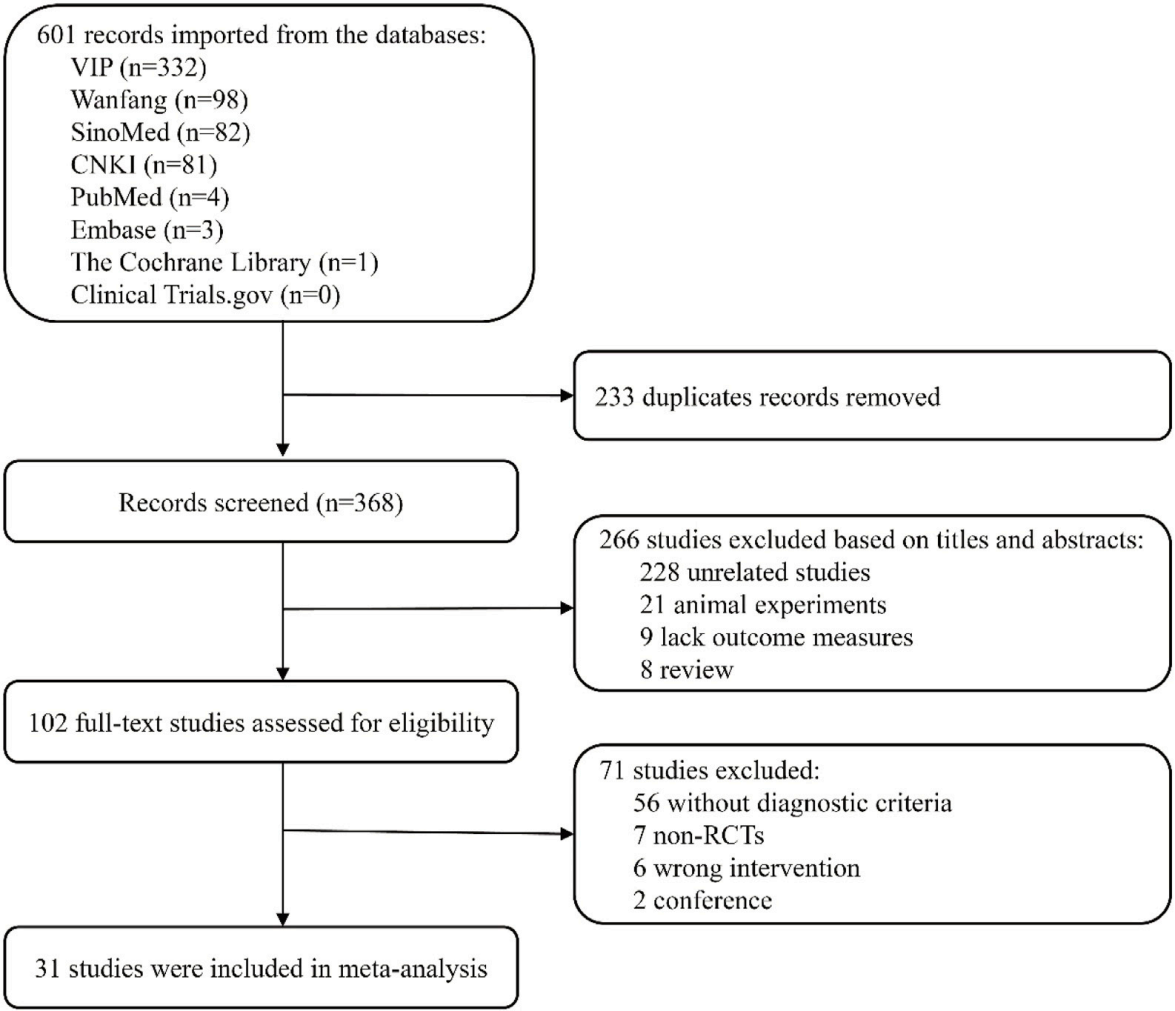
Flowchart of the study search.

A total of 601 relevant studies were screened from the above mentioned databases. Of these, 233 were duplicate studies and 266 studies were excluded based on the title and abstract. After reading the full text, 71 studies were deleted, and ultimately 31 RCTs met the inclusion criteria and were included for the meta-analysis (Figure 1).

### 3.2 Basic characteristics of included RCTs

All included RCTs can be divided into three different comparison measures, namely, SXN vs. placebo, SXN vs. other drugs, and SXN combined with other drugs vs. other drugs. A total of 2,372 patients were included, with an average age between 37.6 and 66.5. The included RCTs were published between 2009 and 2023. The names of other drugs and detailed comparison measures on the inclusion of the study was presented in Table 1.

**TABLE 1:** The detailed characteristics of the included RCTs.

Comparation	First author	Publication year	Type of patients	Group	Age	No.	Intervation	Duration (weeks)
SXN VS other drug	Sun et al. (2022)	2022	DD	Intervation	65.45 ± 11.38	40	SXN	12
				Control	66.31 ± 12.62	40	Ferrlecit 2	
	Zhong et al. (2020)	2020	DD	Intervation	55.68 ± 13.28	38	SXN	12
				Control	50.33 ± 13.25	38	Iron Dextran	
	Tang et al. (2020)	2020	DD	Intervation	58.6 ± 13.4	47	SXN	12
				Control	56.9 ± 10.7	47	Ferrlecit 2	
	Tang et al. (2019)	2019	NDD	Intervation	58.9 ± 14.5	53	SXN	12
				Control	55.1 ± 14.7	41	Ferrlecit 2	
	Shi (2019)	2019	DD	Intervation	47.0 ± 18	15	SXN	8
				Control	49.5 ± 21.5	15	Ferrous Saccharose	
	Xu et al. (2018)	2018	NDD	Intervation	59.3 ± 17.2	35	SXN	12
				Control	56.8 ± 15.7	35	Ferrlecit 2	
	Fan et al. (2017)	2017	DD	Intervation	40.24 ± 17.76	38	SXN	8
				Control	41.37 ± 17.02	34	Ferrous fumarate	
	Zhou et al. (2016a)	2016	DD	Intervation	45.38 ± 9.09	32	SXN	16
				Control	46.06 ± 9.83	32	Ferrlecit 2	
	Zhou et al. (2016b)	2016	DD	Intervation	54.8 ± 3.6	67	SXN	12
				Control	55.3 ± 4.3	67	Iron Dextran	
	Guo (2015)	2015	NDD	Intervation	45.3 ± 19.5	29	SXN	12
				Control		29	Ferrous fumarate	
	Zhang and Xu (2015)	2015	DD	Intervation	45.5 ± 9.22	30	SXN	12
				Control	NA	30	Ferrlecit 2	
	Liu et al. (2015)	2015	DD	Intervation	45.9 ± 18.6	32	SXN	12
				Control	47.1 ± 10.7	32	Ferrlecit 2	
	Wang et al. (2013)	2013	DD	Intervation	45.4 ± 9.2	21	SXN	16
				Control		21	Ferrous Sulfate and Vitamin Complex Sustained-release Tablets	
	Caixia (2013)	2013	DD	Intervation	52.7 ± 11.3	30	SXN	12
				Control		30	Ferrlecit 2	
	Long et al. (2012)	2013	NDD	Intervation	54.2 ± 12	34	SXN	12
				Control	51.6 ± 13.8	34	Ferrous Sulfate	
	Tan et al. (2013)	2013	NA	Intervation	52.7 ± 11.4	58	SXN	12
				Control		58	Ferrous Sulfate	
	Zhang et al. (2010)	2010	NDD	Intervation	37.6 ± 16.4	50	SXN	4
				Control	42.3 ± 13.5	50	ferrous lactate	
	Zhou (2009)	2009	DD	Intervation	NA	77	SXN	8
				Control	NA	77	Iron Dextran	
	Li (2009)	2009	NDD	Intervation	51 ± 3	34	SXN	12
				Control		34	Ferrous Sulfate	
SXN VS Placebo	Zeng and Guo (2022)	2022	DD	Intervation	55.92 ± 4.23	39	SXN	12
				Control	55.86 ± 4.17	39	Placebo	
	Cheng et al. (2016)	2016	DD	Intervation	64.2 ± 6.9	38	SXN	24
				Control	63.5 ± 6.7	34	Placebo	
	Zhang et al. (2016b)	2016	DD	Intervation	48.0 ± 10.9	21	SXN	12
				Control	47.3 ± 10.8	23	Placebo	
	Xu (2012)	2012	DD	Intervation	55.6 ± 11.1	22	SXN	12
				Control		22	Placebo	
	Tang (2011)	2011	DD	Intervation	52.6 ± 10.5	18	SXN	8
				Control		18	Placebo	
SXN + other drug VS other drug	Tang (2023)	2023	NA	Intervation	55.10 ± 4.45	40	SXN + Roxadustat	12
				Control	55.19 ± 4.42	40	Roxadustat	
	Liu et al. (2022)	2022	NA	Intervation	51.22 ± 2.27	49	SXN + Roxadustat	12
				Control	51.24 ± 2.21	50	Roxadustat	
	Huang et al. (2022)	2022	NA	Intervation	40.12 ± 2.60	30	SXN + Roxadustat	8
				Control	40.16 ± 2.63	30	Roxadustat	
	Li et al. (2021)	2021	NDD	Intervation	46.5 ± 13.2	49	SXN + Ferrlecit 2	12
				Control	42.3 ± 10.5	50	Ferrlecit 2	
	Kang and Liang (2020)	2020	NDD	Intervation	46 ± 10.6	28	SXN + Iron Polysaccharide Complex Capsules	4
				Control	45 ± 9.4	26	Iron Polysaccharide Complex Capsules	
	Zeng and Qu (2016)	2016	DD	Intervation	52.35 ± 9.86	47	SXN + Levocarnitine Oral Solution	12
				Control	52.37 ± 9.88	47	Levocarnitine Oral Solution	
	Yi et al. (2015)	2015	NDD	Intervation	52.84 ± 11.5	58	SXN + Iron Polysaccharide Complex Capsules	NA
				Control	52.1 ± 14.0	50	Iron Polysaccharide Complex Capsules	

### 3.3 Risk of bias

Table 2 summarizes the risk of bias for the included studies. Three studies (Cheng et al., 2016; Tang et al., 2020; Liu et al., 2022) were judged as low risk of bias, nine studies (Sun et al., 2022; Zhong et al., 2020; Tang et al., 2019; Zhou et al., 2016a; Zhou et al., 2016b; Liu et al., 2015; Zhang et al., 2010; Zeng and Guo, 2022; Huang et al., 2022) were judged as probably low risk of bias, one study (Zeng and Qu, 2016) was judged as high risk of bias, and the remaining 18 studies (Shi, 2019; Xu et al., 2018; Fan et al., 2017; Guo, 2015; Zhang and Xu, 2015; Wang et al., 2013; Caixia, 2013; Long et al., 2012; Tan et al., 2013; Zhou, 2009; Li, 2009; Zhang W. et al., 2016; Xu, 2012; Tang, 2011; Tang, 2023; Li et al., 2021; Kang and Liang, 2020; Yi et al., 2015) were judged as probably high risk of bias. Overall, the quality of the included studies was not high. However, in the section of “Bias due to missing outcome data,” all studies were judged as low risk of bias and there were no missing outcome data.

**TABLE 2:** The risk of bias assessment in included studies.

Study	Bias arising from the randomization process	Bias due to deviations from the intended intervention	Bias due to missing outcome data	Bias in measurement of the outcome	Bias in selection of the reported results	Other risk of bias	Overall judgement
Sun et al. (2022)	Probably low	Probably low	Low	Probably low	Low	Probably low	Probably low
Zhong et al. (2020)	Probably low	Probably low	Low	Probably low	Low	Probably low	Probably low
Tang et al. (2020)	Low	Low	Low	Low	Low	Probably Low	Low
Tang et al. (2019)	Probably low	Probably low	Low	Probably low	Low	Probably low	Probably low
Shi (2019)	Probably high	Probably low	Low	Probably high	Low	Probably low	Probably high
Xu et al. (2018)	Probably high	Probably low	Low	Probably high	Low	Probably low	Probably high
Fan et al. (2017)	Probably high	Probably low	Low	Probably high	Low	Probably low	Probably high
Zhou et al. (2016a)	Probably low	Probably low	Low	Probably low	Low	Probably low	Probably low
Zhou et al. (2016b)	Probably low	Probably low	Low	Probably low	Low	Probably low	Probably low
Guo (2015)	Probably high	Probably low	Low	Probably high	Probably low	Probably low	Probably high
Cheng et al. (2016)	Probably high	Probably low	Low	Probably high	Low	Probably low	Probably high
Liu et al. (2015)	Probably low	Probably low	Low	Probably low	Low	Probably low	Probably low
Wang et al. (2013)	Probably high	Probably low	Low	Probably high	Probably low	Probably low	Probably high
Caixia (2013)	Probably high	Probably low	Low	Probably low	Probably low	Probably low	Probably high
Long et al. (2012)	Probably high	Probably low	Low	Probably low	Probably low	Probably low	Probably high
Tan et al. (2013)	Probably high	Probably low	Low	Probably high	Probably low	Probably low	Probably high
Zhang et al. (2010)	Probably low	Probably low	Low	Probably low	Probably low	Probably low	Probably low
Zhou (2009)	Probably high	Probably low	Low	Probably low	Probably low	Probably low	Probably high
Li (2009)	Probably high	Probably low	Low	Probably low	Probably low	Probably low	Probably high
Zeng and Guo (2022)	Probably low	Probably low	Low	Probably low	Probably low	Probably low	Probably low
Cheng et al. (2016)	Low	Low	Low	Low	Low	Probably Low	Low
Zhang et al. (2016b)	Probably high	Probably low	Low	Probably low	Probably low	Probably low	Probably high
Xu (2012)	Probably high	Probably low	Low	Probably high	Probably low	Probably low	Probably high
Tang (2011)	Probably high	Probably low	Low	Probably high	Probably low	Probably low	Probably high
Tang (2023)	Probably high	Probably low	Low	Probably high	Probably low	Probably low	Probably high
Liu et al. (2022)	Low	Low	Low	Low	Low	Probably Low	Low
Huang et al. (2022)	Probably low	Probably low	Low	Low	Low	Probably low	Probably low
Li et al. (2021)	Probably high	Probably low	Low	Low	Probably low	Probably low	Probably high
Kang and Liang (2020)	Probably high	Probably low	Low	Low	Probably low	Probably low	Probably high
Zeng and Qu (2016)	High	High	Low	Probably Low	Probably Low	Probably High	High
Yi et al. (2015)	Probably high	Probably low	Low	Probably low	Probably low	Probably low	Probably high

### 3.4 Meta-analysis

#### 3.4.1 Effect of SXN in renal anemia patients

##### 3.4.1.1 Hemoglobin level (primary outcome)

**FIGURE 2:**
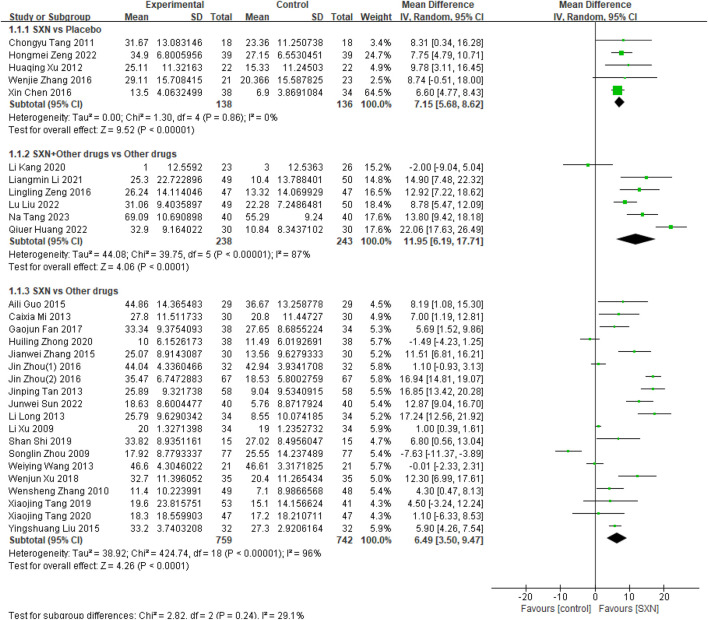
Meta-analysis results on Δ*Hb* after treatment with SXN or SXN + other drugs.

30 included RCTs (Cheng et al., 2016; Sun et al., 2022; Zhong et al., 2020; Tang et al., 2020; Tang et al., 2019; Shi, 2019; Xu et al., 2018; Fan et al., 2017; Zhou et al., 2016a; Zhou et al., 2016b; Guo, 2015; Zhang and Xu, 2015; Liu et al., 2015; Wang et al., 2013; Caixia, 2013; Long et al., 2012; Tan et al., 2013; Zhang et al., 2010; Zhou, 2009; Li, 2009; Zeng and Guo, 2022; Zhang W. et al., 2016; Xu, 2012; Tang, 2011; Tang, 2023; Liu et al., 2022; Huang et al., 2022; Li et al., 2021; Kang and Liang, 2020; Zeng and Qu, 2016) included 2,256 patients reported the changes in Hb from the baseline (Δ*Hb*) after treatment with SXN alone or in combination with other drugs in renal anemia. Among them, 5 RCTs (Cheng et al., 2016; Zeng and Guo, 2022; Zhang W. et al., 2016; Xu, 2012; Tang, 2011) included 274 patients compared Δ*Hb* after treatment with SXN or placebo, 6 RCTs (Tang, 2023; Liu et al., 2022; Huang et al., 2022; Li et al., 2021; Kang and Liang, 2020; Zeng and Qu, 2016) included 481 patients compared the Δ*Hb* after treatment with SXN combined with other drugs or other drugs alone, and 19 RCTs (Sun et al., 2022; Zhong et al., 2020; Tang et al., 2020; Tang et al., 2019; Shi, 2019; Xu et al., 2018; Fan et al., 2017; Zhou et al., 2016a; Zhou et al., 2016b; Guo, 2015; Zhang and Xu, 2015; Caixia, 2013; Long et al., 2012; Tan et al., 2013; Zhang et al., 2010; Zhou, 2009; Li, 2009) included 1,501 patients compared Δ*Hb* after treatment with SXN or other drugs. The meta-analysis results showed that the Δ*Hb* in SXN group was significantly higher than that in placebo group (MD: 7.15 g/L, 95% CI: 5.68–8.62, P < 0.001, the certainty of evidence was high) and other drugs group (MD: 6.49 g/L, 95% CI: 3.50–9.47, P < 0.001, the certainty of evidence was very low). Comprehensive data were presented in Figure 2. The detail of GRADE can be seen in Supplementary Table S1. In addition, the Δ*Hb* in SXN combined with other drugs group was also significantly higher than that in other drugs group (MD: 11.95 g/L, 95% CI: 6.19–17.71, P < 0.001), and the certainty of evidence was moderate (see detail in Supplementary Table S1).

##### 3.4.1.2 Serum ferritin level

**FIGURE 3:**
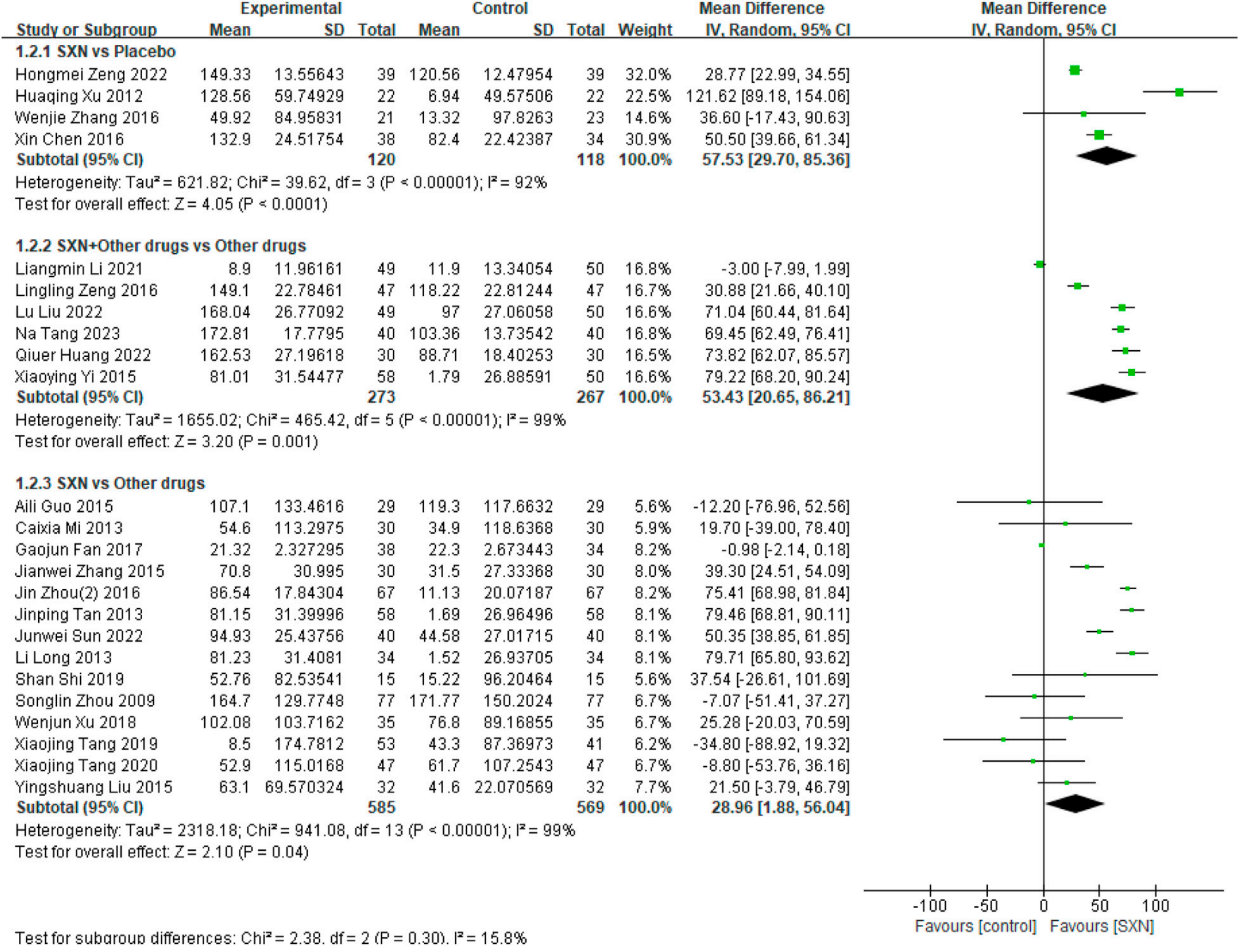
Meta-analysis results on Δ*SF* after treatment with SXN or SXN + other drugs.

24 included RCTs (Cheng et al., 2016; Sun et al., 2022; Tang et al., 2020; Tang et al., 2019; Shi, 2019; Xu et al., 2018; Fan et al., 2017; Zhou et al., 2016b; Guo, 2015; Zhang and Xu, 2015; Liu et al., 2015; Caixia, 2013; Long et al., 2012; Tan et al., 2013; Zhou, 2009; Zeng and Guo, 2022; Zhang W. et al., 2016; Xu, 2012; Tang, 2023; Liu et al., 2022; Huang et al., 2022; Li et al., 2021; Zeng and Qu, 2016; Yi et al., 2015) included 1932 patients reported the change*s in SF from t*he baseline (Δ*SF*) after treatment with SXN alone or in combination with other drugs in renal anemia. Among them, 4 RCTs (Cheng et al., 2016; Zeng and Guo, 2022; Zhang W. et al., 2016; Xu, 2012) included 238 patients compared Δ*SF* after treatment with SXN or placebo, 6 RCTs (Tang, 2023; Liu et al., 2022; Huang et al., 2022; Li et al., 2021; Zeng and Qu, 2016; Yi et al., 2015) included 540 patients compared the Δ*SF* after treatment with SXN combined with other drugs or other drugs alone, and 14 RCTs (Sun et al., 2022; Tang et al., 2020; Tang et al., 2019; Shi, 2019; Xu et al., 2018; Fan et al., 2017; Zhou et al., 2016b; Guo, 2015; Zhang and Xu, 2015; Liu et al., 2015; Caixia, 2013; Long et al., 2012; Tan et al., 2013; Zhou, 2009) included 1,154 patients compared Δ*SF* after treatment with SXN or other drugs. The meta-analysis results showed that the Δ*SF* in SXN group was significantly higher than that in placebo group (MD: 57.53 ng/mL, 95% CI: 29.70–85.36, P < 0.001, the certainty of evidence was moderate) and other drugs group (MD: 28.96 ng/mL, 95% CI: 1.88–56.04, P = 0.04, the certainty of evidence was moderate). Additionally, the Δ*SF* in SXN combined with other drugs group was also significantly higher than that in other drugs group (MD: 53.43 ng/mL, 95% CI: 20.65–86.21, P = 0.001), and the certainty of evidence was moderate. Detailed information can be found in Figure 3.

##### 3.4.1.3 Transferrin saturation level

**FIGURE 4:**
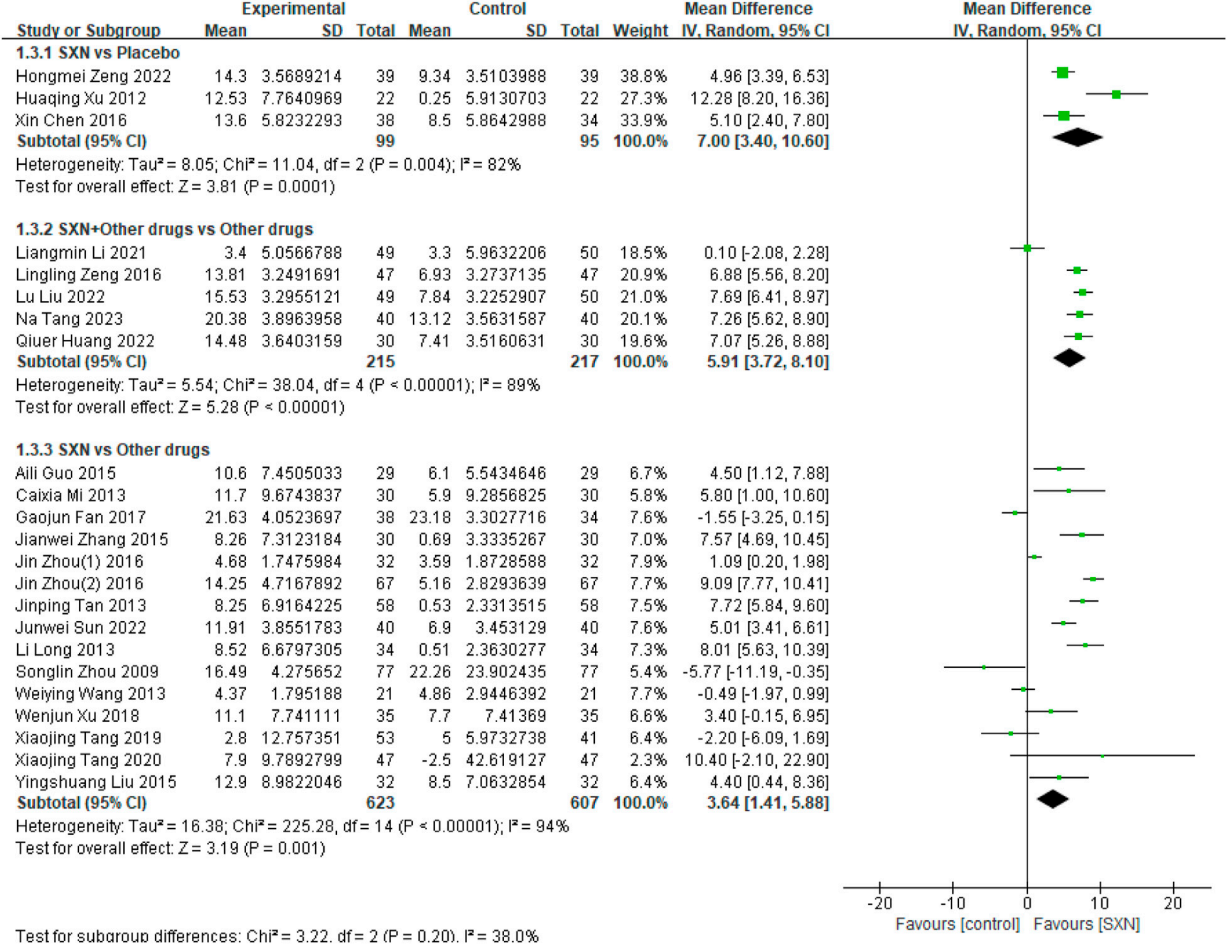
Meta-analysis results on Δ*TSAT* after treatment with SXN or SXN + other drugs.

23 included RCTs (Cheng et al., 2016; Sun et al., 2022; Tang et al., 2020; Tang et al., 2019; Xu et al., 2018; Fan et al., 2017; Zhou et al., 2016a; Zhou et al., 2016b; Guo, 2015; Zhang and Xu, 2015; Liu et al., 2015; Wang et al., 2013; Caixia, 2013; Long et al., 2012; Tan et al., 2013; Zhou, 2009; Zeng and Guo, 2022; Xu, 2012; Tang, 2023; Liu et al., 2022; Huang et al., 2022; Li et al., 2021; Zeng and Qu, 2016) included 1856 patients reported the change*s* in *TSAT* from the baseline (Δ*TSAT*) after treatment with SXN alone or in combination with other drugs in renal anemia. Among them, 3 RCTs (Cheng et al., 2016; Zeng and Guo, 2022; Xu, 2012) included 194 patients compared Δ*TSAT* after treatment with SXN or placebo, 5 RCTs (Tang, 2023; Liu et al., 2022; Huang et al., 2022; Li et al., 2021; Zeng and Qu, 2016) included 432 patients compared the Δ*TSAT* after treatment with SXN combined with other drugs or other drugs alone, and 15 RCTs (Sun et al., 2022; Tang et al., 2020; Tang et al., 2019; Xu et al., 2018; Fan et al., 2017; Zhou et al., 2016a; Zhou et al., 2016b; Guo, 2015; Zhang and Xu, 2015; Liu et al., 2015; Wang et al., 2013; Caixia, 2013; Long et al., 2012; Tan et al., 2013; Zhou, 2009) included 1,230 patients compared Δ*TSAT* after treatment with SXN or other drugs. The meta-analysis results showed that the Δ*TSAT* in SXN group was significantly higher than that in placebo group (MD: 7.00%, 95% CI: 3.40–10.60, P = 0.0001, the certainty of evidence was moderate) and other drugs group (MD: 3.64%, 95% CI: 1.41–5.88, P = 0.001, the certainty of evidence was moderate). Additionally, the Δ*TSAT* in SXN combined with other drugs group was also significantly higher than that in other drugs group (MD: 5.91%, 95% CI: 3.72–8.10, P < 0.001), and the certainty of evidence was moderate. Detailed information can be seen in Figure 4.

##### 3.4.1.4 Serum iron level

**FIGURE 5:**
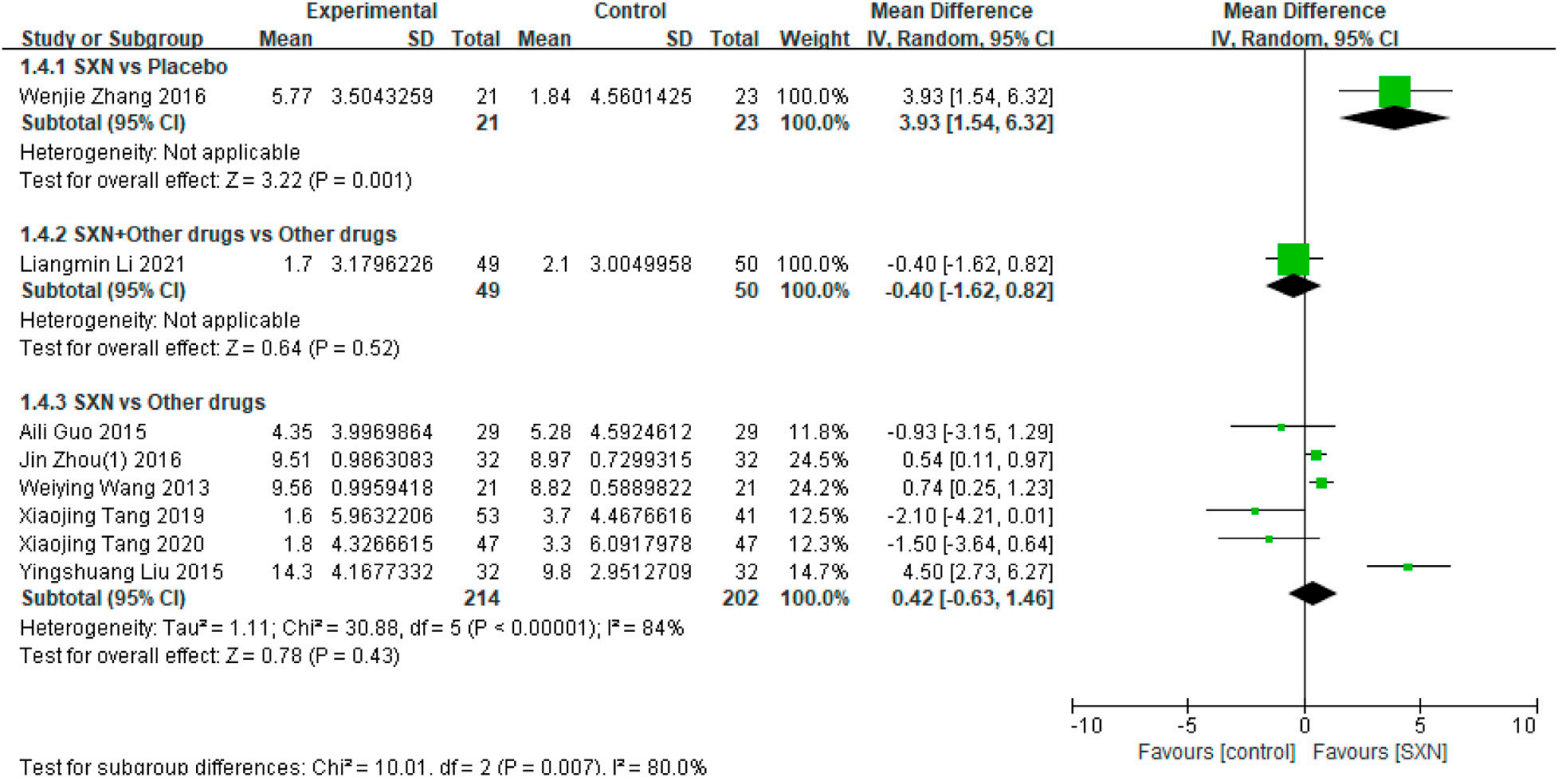
Meta-analysis results on Δ*SI* after treatment with SXN or SXN + other drugs.

Eight included RCTs (Tang et al., 2020; Tang et al., 2019; Zhou et al., 2016a; Guo, 2015; Liu et al., 2015; Wang et al., 2013; Zhang W. et al., 2016; Li et al., 2021) included 559 reported the change*s in SI from t*he baseline (Δ*SI*) after treatment with SXN alone or in combination with other drugs in renal anemia. Among them, one RCTs (Zhang W. et al., 2016) included 44 patients compared Δ*SI* after treatment with SXN or placebo, one RCTs (Li et al., 2021) included 99 patients compared the Δ*SI* after treatment with SXN combined with other drugs or other drugs alone, and 6 RCTs (Tang et al., 2020; Tang et al., 2019; Zhou et al., 2016a; Guo, 2015; Liu et al., 2015; Wang et al., 2013) included 416 patients compared Δ*SI* after treatment with SXN or other drugs. The meta-analysis results showed that the Δ*SI* in SXN group was significantly higher than that in placebo group (MD:3.93 μmol/L, 95% CI: 1.54–6.32, P = 0.001), and the certainty of evidence was low. But there was no significant difference in Δ*SI* between SXN group and other drugs group (MD: 0.42 μmol/L, 95% CI: −0.63–1.46, P = 0.43), the certainty of evidence was moderate. Additionally, the Δ*SI* in SXN combined with other drugs group was also no significant difference in other drugs group (MD: −4.0 μmol/L, 95% CI: −1.62–0.82, P = 0.52), and the certainty of evidence was low. Comprehensive data were presented in Figure 5.

#### 3.4.2 Safety of SXN on the outcomes in renal anemia patients

The common ADRs reported mainly included nausea, constipation, headache, diarrhea, insomnia, abdominal distension, abdominal pain, sore throat, itchy skin, burning sensation in the stomach, and so on. No serious ADRs were reported in all the included RCTs.

##### 3.4.2.1 Adverse drug reactions

**FIGURE 6:**
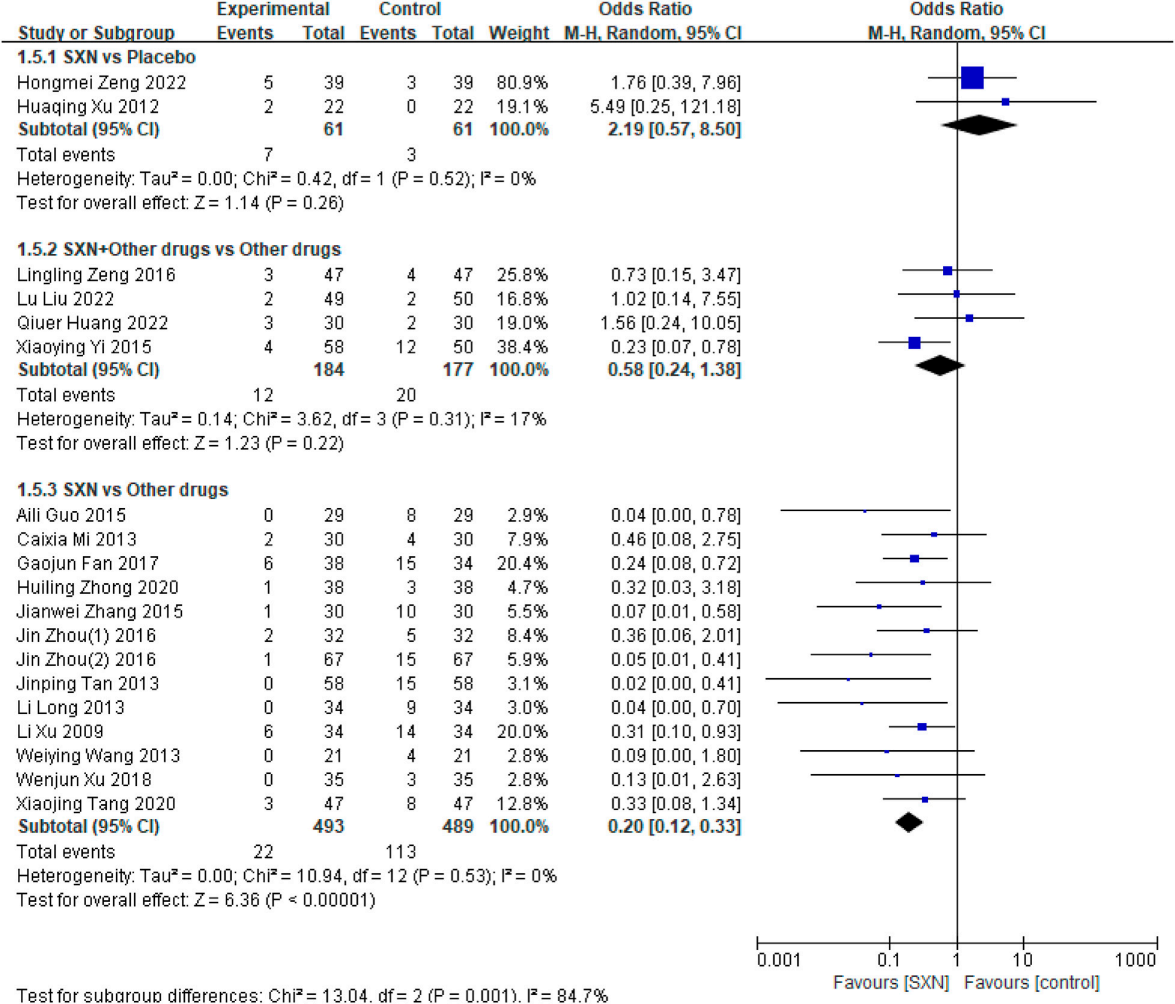
Meta-analysis results on the incidence of ADRs.

ADRs were reported in 19 RCTs (Zhong et al., 2020; Tang et al., 2020; Xu et al., 2018; Fan et al., 2017; Zhou et al., 2016a; Zhou et al., 2016b; Guo, 2015; Zhang and Xu, 2015; Wang et al., 2013; Caixia, 2013; Long et al., 2012; Tan et al., 2013; Li, 2009; Zeng and Guo, 2022; Xu, 2012; Liu et al., 2022; Huang et al., 2022; Zeng and Qu, 2016; Yi et al., 2015) which included 1,465 patients. Among them, two RCTs (Zeng and Guo, 2022; Xu, 2012) included 122 patients compared the incidence of ADRs between SXN- and placebo-treated renal anemia patients, four RCTs (Liu et al., 2022; Huang et al., 2022; Zeng and Qu, 2016; Yi et al., 2015) included 361 patients compared the incidence of ADRs after treatment with SXN combined with other drugs or other drugs alone, and 13 RCTs (Zhong et al., 2020; Tang et al., 2020; Xu et al., 2018; Fan et al., 2017; Zhou et al., 2016a; Zhou et al., 2016b; Guo, 2015; Zhang and Xu, 2015; Wang et al., 2013; Caixia, 2013; Long et al., 2012; Tan et al., 2013; Li, 2009) included 982 patients compared the incidence of ADRs between SXN- and other drugs-treated renal anemia patients. The meta-analysis results showed that there was no significant difference between SXN and placebo group (OR: 2.19, 95%CI: 0.57–8.50, P = 0.26) or between SXN combined with other drugs and other drugs alone group (OR: 0.58, 95%CI: 0.24–1.38, P = 0.22). It is worth noting that the incidence of ADRs in the SXN group was lower than that in the other drugs group (OR: 0.20, 95%CI: 0.12–0.33, P < 0.00001). Detailed information can be seen in Figure 6.

### 3.5 Sensitivity analysis

We conducted a sensitivity analysis to assess the stability and reliability of the meta-analysis results. We used sensitivity analysis on the 31 included studies by excluding studies one by one. The results indicated that excluding any individual study did not significantly alter the therapeutic efficacy of SXN treatment. This suggests that the results of the meta-analysis are stable (see Supplementary Figures S1–S5).

### 3.6 Meta-regression

Due to significant heterogeneity among the included RCTs, we performed meta-regression to identify and analyze the sources of heterogeneity. The observed heterogeneity primarily stems from three sources: (1) variations in intervention protocols, which we categorized into three distinct comparison groups for subgroup analysis in our meta-analysis; (2) differences in patient types, specifically distinguishing between dialysis-dependent (DD) and non-dialysis-dependent (NDD) patient; and (3) variability in the risk of bias across included studies. Based on these factors, we performed meta-regression analyses focusing on patient disease subtypes (named as Type 1) and the risk of bias levels in included studies (named as Type 2). The results demonstrated that neither Type 1 nor Type 2 served as significant sources of heterogeneity among the randomized controlled trials (Table 3), with neither factor compromising the stability of the overall findings.

**TABLE 3:** The results of Meta-regression.

Outcomes	Hb	SF	TSAT	SI	ADR
	P				
Type 1	0.273	0.055	0.121	0.065	0.636
Type 2	0.267	0.265	0.158	0.554	0.115

### 3.7 Publication bias

We found that among the three subgroups, only the comparison between SXN and other drugs included more than 10 studies. In addition, except for the change in SI level, which was reported in less than 10 studies, the number of reports for other outcomes was all greater than 10. Based on this, we conducted an Egger test on the subgroup which compared SXN with other drugs and outcomes included Hb, SF, TSAT, ADR. We found that the P-values from Egger’s test were all greater than 0.05, indicating that there was no publication bias in the results (Table 4).

**TABLE 4:** The results of Egger’s test.

Outcomes	Hb	SF	TSAT	ADR
	P			
Egger	0.696	0.059	0.649	0.774

## 4 Discussion

This study included 31 RCTs and divided them into three subgroups for meta-analysis based on different comparative measures. The results of the study demonstrate that SXN has good efficacy and high safety in treating renal anemia. We found that the results of three subgroups all showed that SXN can effectively increase the levels of Hb, SF, and TSAT in renal anemia, and there were no significant differences in the level of SI between SXN- and other drugs-treated groups. In terms of safety, the incidence of ADRs in the SXN group was significantly lower than that in other drugs group. However, when SXN was combined with other drugs to treat renal anemia, the incidence of ADRs was not different from that in other drugs group.

In our search of the aforementioned databases, we identified two meta-analyses (Zeng et al., 2020; Zhang et al., 2022). The study by Zeng et al. (2020) reported the efficacy and safety of SXN compared with oral iron supplements in the treatment of renal anemia, while Zhang et al. (2022) documented the effectiveness and safety of SXN in treating and preventing iron-deficiency anemia. Our findings are consistent with those of Zeng et al. (2020), confirming that SXN can significantly increase Hb, TSAT, and SF levels, while demonstrating a low incidence of ADRs. Unlike Zeng et al. (2020), our study included a larger sample size than their, rendering the obtained data more persuasive to some extent. In comparison with the study by Zhang et al., 2022), our analysis revealed that SXN can improve Hb and TSAT levels in both renal anemia and iron-deficiency anemia patients. However, the effects of SXN on SF in iron-deficiency anemia patients were not statistically significant. Notably, the incidence of ADRs to SXN in both renal anemia and iron-deficiency anemia patients was lower than that of oral iron supplements, further confirming the safety profile of SXN.

SXN is a second-grade new drug, and its main component, sodium iron chlorophyllin, has a structure similar to heme. It is absorbed by the exclusive channel of heme receptors in small intestinal villous mucosal cells, and is not subject to competitive inhibition by other divalent metal ions or diet, and does not produce free ions or stimulate the gastrointestinal tract (Yin et al., 2013). Sodium iron chlorophyllin can also improve iron metabolism, promote iron absorption, increase the release of stored iron in the body, increase the level of SI and TSAT, promote hematopoiesis, and improve the condition of renal anemia (Suryavanshi et al., 2015; Ouameur et al., 2005). It has the characteristics of high bioavailability and good regulatory performance. In addition, SXN can improve the micro inflammatory state, reduce the levels of C-reactive protein, tumor necrosis factor, and interleukin-6 (Liu and Hu, 2016).

At present, the drugs used to treat renal anemia include oral iron supplements and intravenous iron supplements. Generally speaking, intravenous iron injection is superior to oral supplements. However, when using intravenous iron injection, we should fully consider the possible adverse reactions, including allergic reactions, infections, and oxidative stress (Bansal et al., 2014; Akarsu et al., 2013). Several studies had found that micro-inflammation and oxidative stress can increase the incidence and mortality of cardiovascular events (Rozen-Zvi et al., 2008; Martinez-Vea et al., 2012). Although oral iron is easy to use and cost-effective, its efficacy is not as good as intravenous iron injection. In addition, oral iron supplements can cause adverse gastrointestinal reactions such as constipation, loss of appetite, bloating, and diarrhea (Tolkien et al., 2015; Kortman et al., 2017), the frequency of gastrointestinal-related side effects can impact patients’ compliance with iron treatment (Lewkowitz and Tuuli, 2019). SXN is an efficient iron supplement with a high absorption rate of 25%–30%, which is 12.5 times higher than other oral iron supplements. It can also promote the absorption of other free iron in food (Jiahuan et al., 2017). SXN is superior to other oral iron supplements in many aspects. Its main component, sodium iron chlorophyllin, remains stable under gastrointestinal conditions, avoiding the common gastrointestinal adverse reactions of traditional oral iron supplements (Ding et al., 2019; Miret et al., 2010), and rarely associated with ADRs (Sheng-Ming et al., 2018).

The study has several limitations: First, we conducted three subgroup analyses, but heterogeneity still exists in several outcomes. This may be due to the fact that some patients in the included studies require dialysis treatment, while others do not. However, we conducted a meta-regression on both types of patients and found that different patient types did not affect the heterogeneity between the studies. We speculate that heterogeneity may be caused by the differences in the number of patients and treatment durations in the included studies; Second, of the 31 studies included, only three RCTs were accessed as a low risk of bias, while 18 were accessed as a high risk of bias. Therefore, the methodological quality of the included RCTs may be low; Third, although we conducted a comprehensive search and used GRADE to evaluate the quality of evidence in order to ensure that all relevant studies were included and reduce the possibility of publication bias, we found that due to the low quality of the main studies or insufficient trials, the overall level of evidence was not high, and evidence users needed to be careful.

## 5 Conclusion

According to the meta-analysis results of 31 RCTs, the efficacy of SXN in treating renal anemia is convincing. Compared with other drugs, SXN is comparable or even better in treating renal anemia. Additionally, the safety of SXN is also relatively high. We hope that further researches can overcome the limitations of this study and provide stronger evidence for the effectiveness and safety of SXN, and bring more treatment possibilities to patients with renal anemia.

## Data Availability

The original contributions presented in the study are included in the article/Supplementary Material, further inquiries can be directed to the corresponding author.
