# Supplementary material for: Effectiveness and safety of Shengxuening for treatment of renal anemia: a comprehensive systematic review and meta-analysis
Source: Front Pharmacol. 2025 Jun 24;16:1510227. doi: 10.3389/fphar.2025.1510227 (PMC12235605; doi:10.3389/fphar.2025.1510227)
Supplement: Supplementary file 1 [file Supplementaryfile1.docx]

**Text S1: Search strategies for all databases**

1. **the China Knowledge Network**

((SU='生血宁' OR SU='生血宁片' OR SU='蚕砂提取物' OR SU='铁叶绿酸钠') OR (TI='生血宁' OR TI='生血宁片' OR TI='蚕砂提取物' OR TI='铁叶绿酸钠')) AND ((SU='肾' AND SU='贫血') OR (SU='肾性贫血' OR SU='肾贫血' OR SU='肾脏贫血'))

1. **the Wanfang Data Knowledge Service Platform**

(题名或关键词: “生血宁” or “生血宁片” or “蚕砂提取物” or “铁叶绿酸钠”) and ((题名或关键词: “肾” and “贫血”) or (题名或关键词: “肾性贫血” or “肾贫血” or “肾脏贫血”))

1. **Technology Journal Database**

(M=生血宁 OR M=生血宁片 OR M=蚕砂提取物 OR M=铁叶绿酸钠) AND ((M=肾 AND M=贫血) OR (M=肾性贫血 OR M=肾贫血 OR M=肾脏贫血))

1. **SinoMed**

#1 "生血宁"[不加权:扩展] OR "生血宁片"[不加权:扩展] OR "蚕砂提取物"[不加权:扩展] OR "铁叶绿酸钠"[不加权:扩展]

#2 "生血宁"[常用字段:智能] OR "生血宁片"[常用字段:智能] OR "蚕砂提取物"[常用字段:智能] OR "铁叶绿酸钠"[常用字段:智能]

#3 #1 OR #2

#4 "肾性贫血"[常用字段:智能] OR "肾贫血"[常用字段:智能] OR "肾脏贫血"[常用字段:智能]

#5 #3 AND #4

1. **PubMed**

#1 renal anemia*[Title/Abstract] OR kidney anemia*[Title/Abstract]

#2 "shengxuening" [Supplementary Concept]

#3 shengxuening [Title/Abstract]

#4 #2 OR #3

#5 "Randomized Controlled Trial"[Publication Type] OR "Controlled Clinical Trial"[Publication Type] OR "Clinical Trials as Topic"[Mesh] OR randomized [Title/Abstract] OR randomized [Title/Abstract] OR randomly [Title/Abstract] OR placebo [Title/Abstract] OR trial [Title]

#6 #1 AND #4 AND #5

1. **Embase**

#1 'renal anemia':ab,ti OR 'kidney anemia*':ab,ti

#2 shengxuening:ab,ti

#3 'randomized controlled trial':it OR 'controlled clinical trial':it OR 'clinical trials as topic'/exp/mj OR randomized:ab,ti OR randomly:ab,ti OR placebo:ab,ti OR trial:ti

#4 #1 and #2 and #3

1. **Cochrane Central Register of Controlled Trials**

#1 MeSH descriptor: [renal anemia] explode all trees

#2 ("shengxuening"):ti,ab,kw

#3 #1 AND #2

1. **Clinical Trials.gov**

Condition or disease: renal anemia

Intervention/Treatment: shengxuening

**Table S1**

PICO (1.1)

Population: renal anemia

Intervention: SXN or SXN+Other drugs

Comparator: Placebo or other drugs

**Table S1 Summary of findings of GRADE assessment**

| Outcome | Outcome | Study results and measurements | Absolute effect estimates | | Certainty of the evidence | Summary |
| --- | --- | --- | --- | --- | --- | --- |
|  | Timeframe |  | other drugs | SXN | (Quality of evidence) |  |
| ΔHb | ΔHb-SXN vs Placebo | Measured by: | Difference: MD 7.15 higher | | High | Compared with placebo, SXN may increase Hb. |
|  |  | Scale: - High better |  |  |  |  |
|  |  | Based on data from 274 participants in 5 studies |  |  |  |  |
|  |  |  | (CI 95% 5.68 lower - 8.62 higher) | |  |  |
|  |  |  |  |  |  |  |
|  | ΔHb-SXN+Other drugs vs Other drugs | Measured by: | Difference: MD 11.95 higher | | Moderate | Compared with Other drugs, SXN+Other drugs may increase Hb. |
|  |  | Scale: - High better |  |  | Due to serious inconsistency^1^ |  |
|  |  | Based on data from 481 participants in 6 studies |  |  |  |  |
|  |  |  | (CI 95% 6.19 lower - 17.71 higher) | |  |  |
|  |  |  |  |  |  |  |
|  | ΔHb-SXN vs Other drugs | Measured by: | Difference: MD 6.49 higher | | Very low | Compared with Other drugs, we are uncertain whether SXN increases or decreases Hb. |
|  |  | Scale: - High better |  |  | Due to serious inconsistency, Due to very serious inconsistency, Due to serious inconsistency, Due to very serious inconsistency, Due to serious imprecision^2^ |  |
|  |  | Based on data from 1501 participants in 19 studies |  |  |  |  |
|  |  |  | (CI 95% 3.50 lower - 9.47 higher) | |  |  |
|  |  |  |  |  |  |  |
| ΔSF | ΔSF-SXN vs Placebo | Measured by: | Difference: MD 57.53 higher | | Moderate | Compared with Placebo, SXN may increase SF. |
|  |  | Scale: - High better |  |  | Due to serious inconsistency, Due to serious imprecision^3^ |  |
|  |  | Based on data from 238 participants in 4 studies |  |  |  |  |
|  |  |  | (CI 95% 29.70 lower - 85.36 higher) | |  |  |
|  |  |  |  |  |  |  |
|  | ΔSF-SXN+Other drugs vs Other drugs | Measured by: | Difference: MD 53.43 higher | | Moderate | Compared with Other drugs, SXN+Other drugs may increase SF. |
|  |  | Scale: - High better |  |  | Due to very serious inconsistency, Due to serious inconsistency^4^ |  |
|  |  |  |  |  |  |  |
|  |  |  | (CI 95% 20.65 lower - 86.21 higher) | |  |  |
|  |  |  |  |  |  |  |
|  | ΔSF-SXN vs Other drugs | Measured by: | Difference: MD 28.96 higher | | Moderate | Compared with Other drugs, SXN may increase SF. |
|  |  | Scale: - High better |  |  | Due to serious inconsistency, Due to serious indirectness^5^ |  |
|  |  | Based on data from 1154 participants in 14 studies |  |  |  |  |
|  |  |  | (CI 95% 1.88 lower - 56.04 higher) | |  |  |
|  |  |  |  |  |  |  |
| ΔTSAT | ΔTSAT-SXN vs Placebo | Measured by: | Difference: MD 7.00 higher | | Moderate | Compared with Placebo, SXN may increase TSAT. |
|  |  | Scale: - High better |  |  | Due to serious inconsistency^6^ |  |
|  |  | Based on data from 194 participants in 3 studies |  |  |  |  |
|  |  |  | (CI 95% 3.40 lower - 10.60 higher) | |  |  |
|  |  |  |  |  |  |  |
|  | ΔTSAT-SXN+Other drugs vs Other drugs | Measured by: | Difference: MD 5.91 higher | | Moderate | Compared with Other drugs, SXN+Other drugs may increase TSAT. |
|  |  | Scale: - High better |  |  | Due to serious inconsistency^7^ |  |
|  |  | Based on data from 432 participants in 5 studies |  |  |  |  |
|  |  |  | (CI 95% 3.72 lower - 8.10 higher) | |  |  |
|  |  |  |  |  |  |  |
|  | ΔTSAT-SXN vs Other drugs | Measured by: | Difference: MD 3.64 higher | | Moderate | Compared with Other drugs, SXN may increase TSAT. |
|  |  | Scale: - High better |  |  | Due to serious risk of bias, Due to serious inconsistency^8^ |  |
|  |  | Based on data from 1230 participants in 15 studies |  |  |  |  |
|  |  |  | (CI 95% 1.41 lower - 5.88 higher) | |  |  |
|  |  |  |  |  |  |  |
| ΔSI | ΔSI-SXN vs Placebo | Measured by: | Difference: MD 3.93 higher | | Low | Compared with Placebo, SXN may may have little in improve SI level. |
|  |  | Scale: - High better |  |  | Due to serious risk of bias, Due to serious imprecision^9^ |  |
|  |  | Based on data from 44 participants in 1 studies |  |  |  |  |
|  |  |  | (CI 95% 1.54 lower - 6.32 higher) | |  |  |
|  |  |  |  |  |  |  |
|  | ΔSI-SXN+Other drugs vs Other drugs | Measured by: | Difference: MD 0.40 lower | | Low | Compared with other drugs, SXN+Other drugs may have no difference in improve SI level. |
|  |  | Scale: - High better |  |  | Due to serious risk of bias, Due to serious imprecision^10^ |  |
|  |  | Based on data from 99 participants in 1 studies |  |  |  |  |
|  |  |  | (CI 95% -1.62 lower - 0.82 higher) | |  |  |
|  |  |  |  |  |  |  |
|  | ΔSI-SXN vs Other drugs | Measured by: | Difference: MD 0.42 higher | | Moderate | Compared with other drugs, SXN have no difference in improve SI level. |
|  |  | Scale: - High better |  |  | Due to serious inconsistency^11^ |  |
|  |  | Based on data from 416 participants in 6 studies |  |  |  |  |
|  |  |  | (CI 95% 0.63 lower - 1.46 higher) | |  |  |
|  |  |  |  |  |  |  |

1. Inconsistency: serious. The magnitude of statistical heterogeneity was high, with I^2:... %.;
2. Inconsistency: very serious. The magnitude of statistical heterogeneity was high, with I^2:... %., Point estimates vary widely; Imprecision: serious. Wide confidence intervals;
3. Inconsistency: serious. The magnitude of statistical heterogeneity was high, with I^2:... %.;
4. Inconsistency: serious. The magnitude of statistical heterogeneity was high, with I^2:... %.;
5. Inconsistency: serious. The magnitude of statistical heterogeneity was high, with I^2:... %.;
6. Inconsistency: serious. The magnitude of statistical heterogeneity was high, with I^2:... %.;
7. Inconsistency: serious. The magnitude of statistical heterogeneity was high, with I^2:... %.;
8. Inconsistency: serious. The magnitude of statistical heterogeneity was high, with I^2:... %.;
9. Risk of Bias: serious. Inadequate sequence generation/ generation of comparable groups, resulting in potential for selection bias, Inadequate/lack of blinding of participants and personnel, resulting in potential for performance bias; Imprecision: serious. Only data from one study;
10. Risk of Bias: serious. Inadequate concealment of allocation during randomization process, resulting in potential for selection bias; Imprecision: serious. Only data from one study;
11. Inconsistency: serious. The magnitude of statistical heterogeneity was high, with I^2:. %.;

**Figure S1-S5 The results of sensitivity analysis**

Figure S1. The sensitivity analysis result of Hb

Figure S2. The sensitivity analysis result of SF

Figure S3. The sensitivity analysis result of TSAT

Figure S4. The sensitivity analysis result of SI

Figure S5. The sensitivity analysis result of ADRs
